# Organic Molecule Detection Based on SERS in Microfluidics

**DOI:** 10.1038/s41598-019-53478-7

**Published:** 2019-11-27

**Authors:** Xin Zhang, Haiyan Zhang, Sheng Yan, Zugang Zeng, Anshou Huang, Anping Liu, Yuan Yuan, Yingzhou Huang

**Affiliations:** 1 Chongqing Key Laboratory of Soft Condensed Matter Physics and Smart Materials, College of Physics, Chongqing University, Chongqing, 400044 China; 2 Chongqing Environment & Sanitation Group Co., LTO, Chongqing, 401121 China; 3 Chongqing Industry Polytechnic College, Chongqing, 400044 China; 4 Department of Chemistry, University of Tokyo, Tokyo, Japan

**Keywords:** Nanophotonics and plasmonics, Environmental monitoring

## Abstract

Sensitive *in situ* detection of organic molecules is highly demanded in environmental monitoring. In this work, the surface enhanced Raman spectroscopy (SERS) is adopted in microfluidics to detect the organic molecules with high accuracy and high sensitivity. Here the SERS substrate in microchannel consists of Ag nanoparticles synthesized by chemical reduction. The data indicates the fabrication conditions have great influence on the sizes and distributions of Ag nanoparticles, which play an important role on the SERS enhancement. This result is further confirmed by the simulation of electromagnetic field distributions based on finite difference time domain (FDTD) method. Furthermore, the SERS spectra of organic molecule (methylene blue) obtained in this plasmonic microfluidic system exhibit good reproducibility with high sensitivity. By a combination of SERS and microfluidics, our work not only explores the research field of plasmonics but also has broad application prospects in environmental monitoring.

## Introduction

Raman spectroscopy is a well-known analytic tool, which can be used for molecule detection without labeling and destructiveness of objects^1,2^. However, the Raman intensity becomes weak when detecting tiny amounts of target molecules. To overcome this issue, surface-enhanced Raman scattering (SERS) has been developed to improve the sensitivity of Raman detection^3^, which can provide an ultra-sensitive spectral analysis of probing molecules. Beyond the metal-like plasmonic materials (e.g., silicon sandwich, graphene, and titanium nitride), noble metals (Au, Ag, Cu) with coarse surface are active SERS substrate^4–10^. Among these noble metals, Ag exhibits the best optical absorption and scattering properties due to localized surface plasmon resonance (LSPRs)^11,12^. The huge electromagnetic field near the surface of noble metals is a major enhanced factor in Raman intensity, which can detect molecules in ultra-low concentration solutions^13–16^. SERS has been explored in many research fields of environmental science^17^, medicine^18^, and biology^19,20^.

According to the measurement format, the SERS detection can be classified into static solid measurement, static liquid measurement and dynamic liquid measurement^21^. Among these measurement methods, the dynamic liquid measurement offers more reliable and reproducible results. The SERS, integrated with microfluidics that can temporally and spatially control the liquid, enables the dynamic liquid measurement^21^. To fabricate SERS-integrated microfluidic devices, different methods have been used to fabricate the SERS substrate within the microfluidic channel. For example, Carboni *et al*. used chemical method to synthesize Ag nanoparticles (NPs) in a microfluidic channel^22^. In addition, Leem *et al*. used a polyol method to heat a microfluidic device to synthesize an Ag film substrate in a microfluidic channel with continuously injecting Ag precursors^23^. However, the NPs synthesized by these methods are non-uniform due to the flow of precursors in the microfluidic channels, and the Raman signal of the molecule can only be greatly enhanced at a certain position. Therefore, it is necessary to fabricate a uniform SERS substrate. It has been reported in the literature that polyols can be used as metal ion solvents and reducing agents, and the process is usually carried out by heating at 90–160 °C to increase the speed of NP synthesis^24^. In this process, the addition of polyvinyl pyrrolidone (PVP) can achieve protection of NPs^25^, preventing AgNPs agglomeration^26^ and morphological selective action^27^. In addition, Cu^2+^ can promote the formation of NP crystallization^28^.

In this paper, the SERS substrate is synthesized in a microfluidic device at low temperature of 150 °C. The injection is stopped when the Ag precursor is filled in the entire channel, and then the microfluidic device is uniformly heated on the heating plate. The even AgNPs are formed on the heated surfaces of the substrate. Then, the Ag precursor is introduced into the microfluidic channel multiple times and heated several times to produce AgNPs as a better SERS active substrate. Therefore, the preparation method has two important advantages: (1) it provides a simple method for preparing AgNPs in a microfluidic channel and (2) this method can synthesize a uniform SERS-active substrate in a microfluidic channel.

## Results and Discussion

### The fabrication of SERS substrate in a microfluidic channel

**Figure 1:**
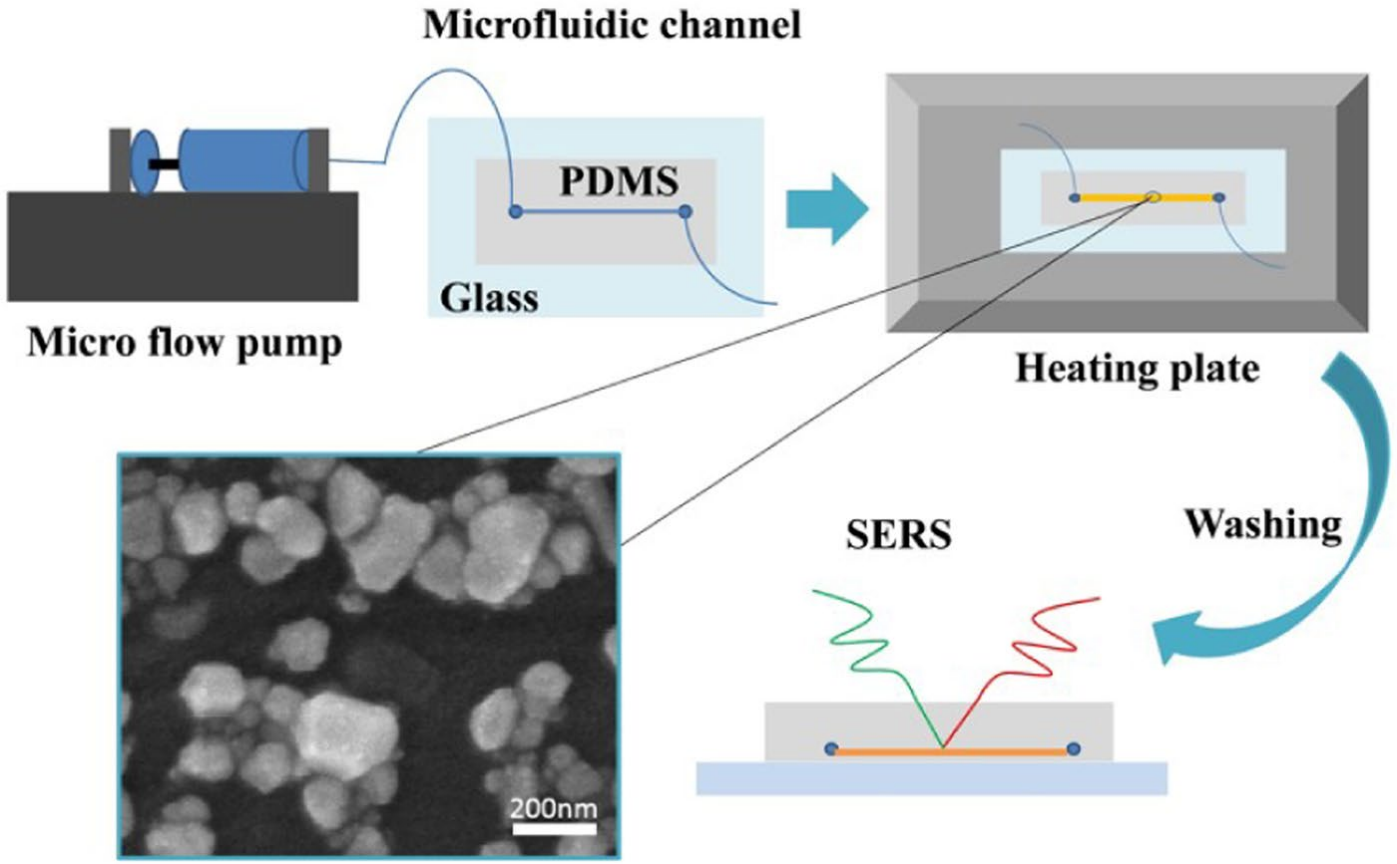
Diagram of the microfluidic patterning system. First, the microfluidic channel is filled with the silver precursor and then stopped. Next, the PDMS channel was heated at 150 °C on a heating plate. Then, wash the channel with alcohol. Finally, SERS detection is performed. The SEM image shows the AgNPs produced in the microfluidic channel.

The experimental process of this work is shown in Fig. 1. The Ag precursor was prepared as followed: AgNO_3_ in EG, CuCl_2_ in EG, and PVP in EG were mixed. The Ag precursor was injected into the microfluidic channel by a syringe pump at a rate of 10 μL min^−1^, and then the microfluidic tube filled with the precursor was placed on a hot plate (150 °C) to react. As reported, the enhancement effect of the SERS substrate is affected by the PVP of the surface of the AgNPs. Therefore, after stopping the heating, we slowly rinsed the PVP on the surface of the Ag particles with alcohol without affecting the synthesized AgNPs. Due to the limited number of Ag atoms injected, the synthesized AgNPs were small and loose. To generate the dense nanoparticles, the same amount of Ag precursor will continue to be injected. Then, the above experimental procedures were repeated to deposit AgNPs on the original AgNPs. Finally, more and larger AgNPs were synthesized. In this way, the disadvantage of uneven substrate is avoided. Normally, with the continuous injection of Ag precursor, the size and density of the AgNPs that are far away from the inlet will slowly decrease. It makes the synthesized SERS substrate uneven, and only effectively enhances the SERS signal of the target molecule in a certain place, but the weaker SERS signals can be obtained in other places^23,29^. The reason may be that many Ag atoms synthesize AgNPs at the entrance. As the liquid flows, the amount of Ag ions is less away from the entrance of the microfluidic channel, resulting in smaller and thinner synthetic AgNPs. In this work, the amount of Ag ions in the microfluidic channel is the same anywhere, and then by heating, a relatively uniform SERS substrate can be obtained. Finally, the methylene blue is injected as probe molecules to detect the performance of the SERS substrate.

### The variation in the morphology of the AgNPs and SERS single with the duration of fabrication

**Figure 2:**
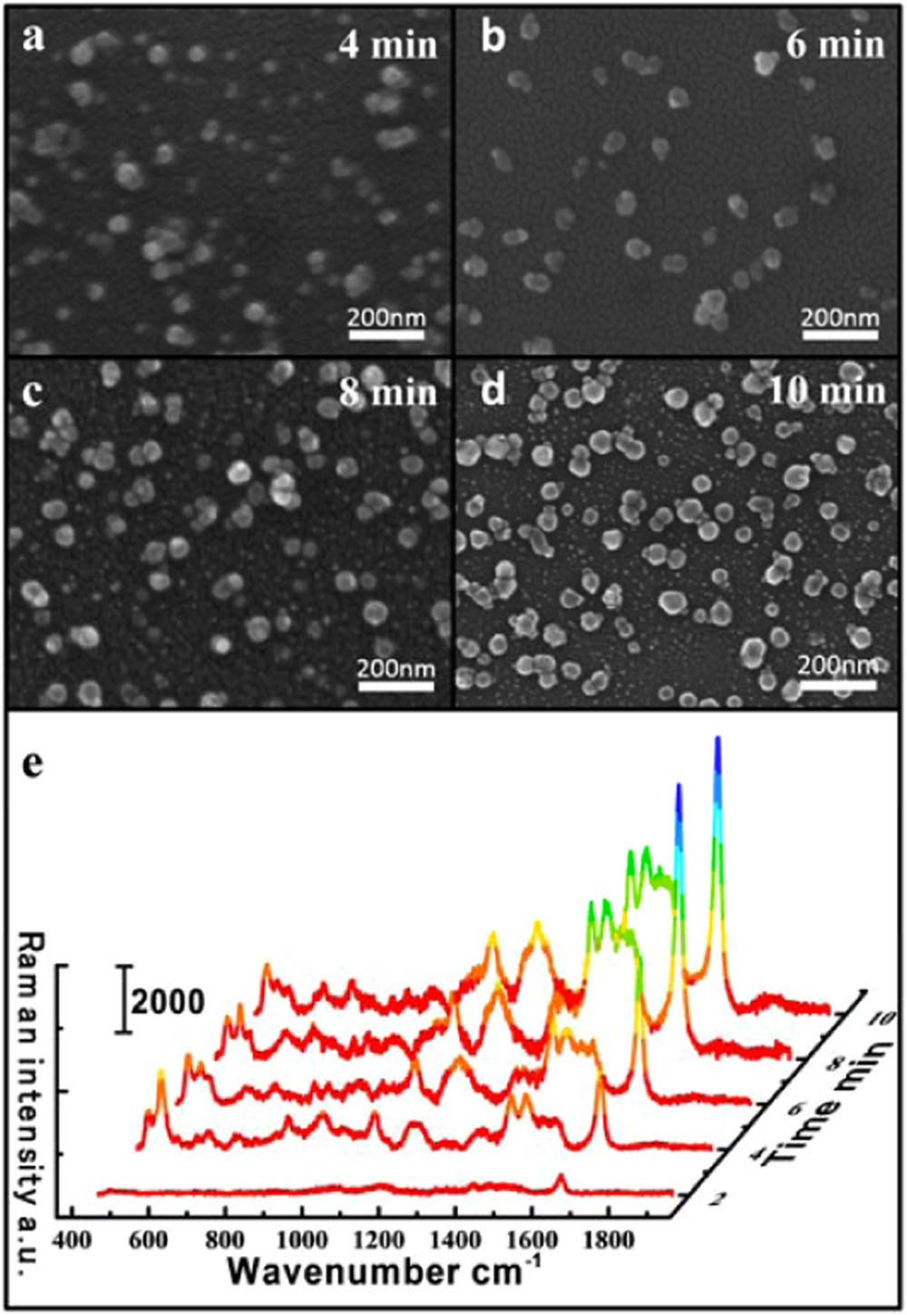
(**a–d**) SEM images showing the variation in the morphologies of the AgNPs with fabrication durations of 4 minutes, 6 minutes, 8 minutes, 10 minutes. (**e**) Raman intensity of MB with different fabrication duration of AgNPs in the channel.

To obtain the optimal reaction time to fabricate the SERS substrate, we tested the device with different duration. The AgNO_3_ solution was injected into the microfluidic channel at a constant rate and concentration. To determine the optimal heating duration of the SERS substrate, various heating durations were prepared: 2 minutes, 4 minutes, 6 minutes, 8 minutes and 10 minutes, and then injected into the target molecule for Raman detection of the microfluidic SERS substrate. The target molecule is MB (1 × 10^−5^ M). Figure 2e shows the SERS spectra of MB in various heating durations. As the heating duration is accumulated, the intensity of the Raman peak of the molecule is also greatly enhanced. After 8 minutes, the intensity of the Raman peak was not significantly enhanced, and the intensity of the Raman peak was approximately the same as that of the Raman peak heated for 8 minutes.

To characterize the synthesized NPs, we photographed the morphology of AgNPs generated on the microfluidic channel using emission scanning electron microscopy (SEM). Without destroying the Ag substrate, we first stripped the PDMS channel with a knife and observed the morphology by SEM. To give a brief overview of the general growth of the SERS substrate, we only show the SEM images of the AgNPs for 4 minutes, 6 minutes, 8 minutes and 10 minutes, as shown in Fig. 2a–d The result suggests that the AgNPs becomes denser as the heating duration increases. It is reported in the literature that the local electromagnetic field at the gap between the NPs becomes stronger as the gap decreases between particles. This results in a significant increase in the Raman signal of the molecules, which are at the gaps of the nanoparticles^29^. This can explain that the Raman signal of the molecule increases with the accumulation of heating duration. Comparing Fig. 2c,d, the Ag substrate produced in 8 minutes is similar to the Ag substrate in 10 minutes. So at 10 minutes, the intensity of the molecule no longer continues to increase dramatically. The reason is that the capacity of the Ag precursor that we heat each duration is constant, which makes the number of Ag ions constant. After heating for 8 minutes, no more Ag ions can participate in the reaction to synthesize AgNPs, and even if heating is continued, the Ag substrate does not change significantly. In summary, in order to save the reaction duration of the sample without affecting the detection sensitivity of the substrate, we chose the 8 minutes as a single heating duration.

### The variation in the morphology of the AgNPs and SERS single with the heating times of fabrication

**Figure 3:**
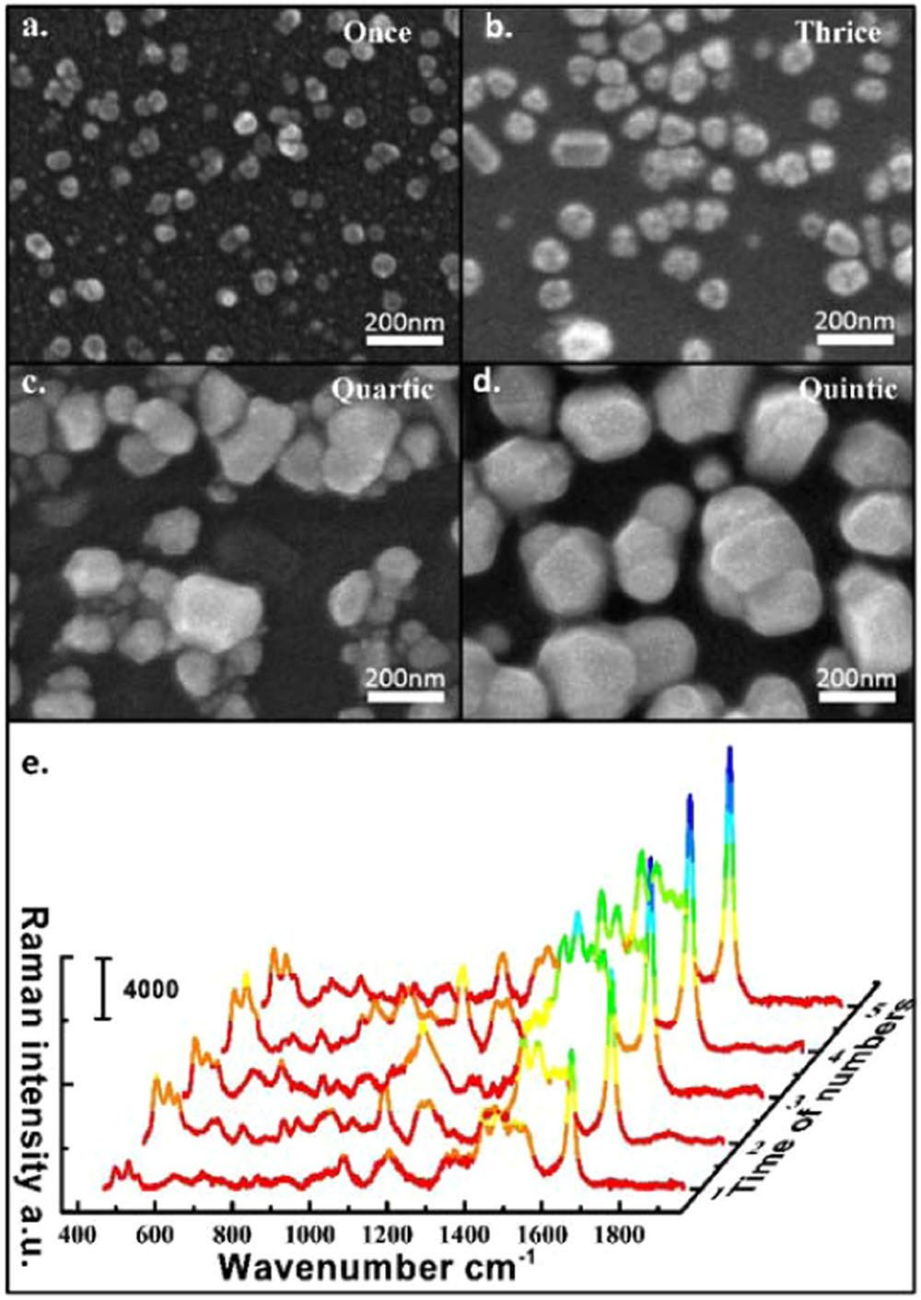
(**a–d**) SEM images showing the variation in the morphologies of the AgNPs with heating times of once, thrice, quartic, quantic. (**e**) Raman intensity of MB with different heating times of AgNPs in the channel.

In the above experiment, a stronger SERS-active substrate having strong SERS activity was obtained. However, the microfluidic channel can only inject a very small amount of Ag precursor, which results in a smaller amount of Ag ions. Even if Ag ions are all reacted to synthesize AgNPs, the obtained AgNPs are smaller, which results in a larger gap between the NPs. To further improve the sensitivity of the substrate, we inject the Ag precursors again, and then heat them to continue the reaction of Ag ions on the original substrate to synthesize Ag atoms, so that the diameter of the AgNPs will continue to increase. Since the volume of the microfluidic channel is constant, this results in a decrease in the gap between the AgNPs as the diameter of the AgNPs increases. We tested the SERS substrate with different heating times of Ag precursors (from top: once, twice, thrice, quartic and quintic). Figure 3e shows the SERS spectra of MB in various heating times. The intensity is gradually increased with the increase of heating time, but after four times, the increase in strength is not significant. To demonstrate the intensity will gradually increase until it tends to stabilize with the increase in the number of heating.

To briefly explain the growth, four substrates were fabricated over various heating times: once, thrice, quartic and quantic. Figure 3a–d show SEM images of the resulting AgNPs. The size of the AgNPs increases and the gap between the NPs will decrease as the heating times. To a certain extent, the Raman intensity of the molecule is stronger as the gap of the NPs becomes smaller. However, when heated five times, some of the NPs that are closer together will slowly grow together and form a whole gradually. The gap between the NPs was not greatly reduced as compared with the substrate obtained by heating four times. This shows that the Raman intensity of the molecules on the substrate heated five times is not significantly enhanced compared to the Raman intensity on the substrate heated four times. In the experiment, the microfluidic SERS substrate is produced by injecting four Ag precursors by heating without affecting the detection sensitivity and saving the raw materials and reducing the production time.

### FDTD software simulates the electric field distribution of Ag nano-dimers with different sizes and different gaps

**Figure 4:**
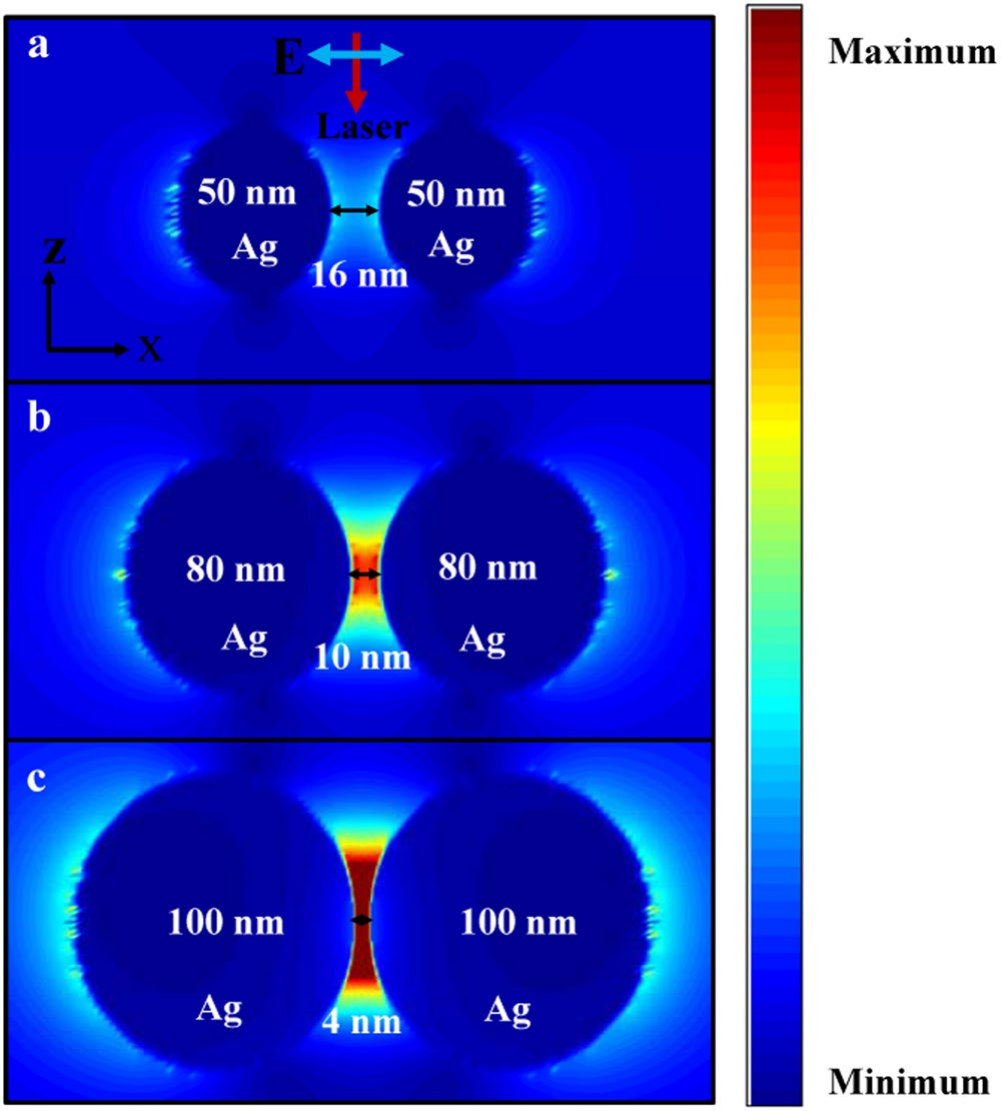
(**a–c**) The FDTD simulation shows the electric field distribution of Ag nano-dimers with different sizes and different gaps.

The experimental results above show that different SERS substrates can be made by increasing the heating duration and heating times. Within a certain range, the activity of the substrate becomes stronger as the heating time was lengthened, and the number of heating times was increased. Theoretically, we explored the phenomena occurring in the above experiments. We used FDTD software to briefly simulate the electric field distribution of Ag nano-dimers with different sizes and different gaps. To briefly explain the reduction of the gap between AgNPs, which affected the SERS activity of the substrate, we simulated the electric field distribution of the two particles by changing the size and gap of the two particles, as shown in Fig. 4a–c. As the gap of the NPs decreases, the local electric field between the NPs becomes stronger. The reason is that the larger free electrons are accumulated near the gap between the two NPs as the gap between the NPs decreases. This causes the local electric field to become stronger at the NP gap^15,30–32^. This is consistent with the results reported in previous work. When the molecules are at these gaps, their Raman signals are greatly enhanced^12^. This is the reason why the substrate obtained by heating for a long time and heating times has a better SERS activity.

### The SERS map of MB from different collection positions and the lowest detection concentration of the device

**Figure 5:**
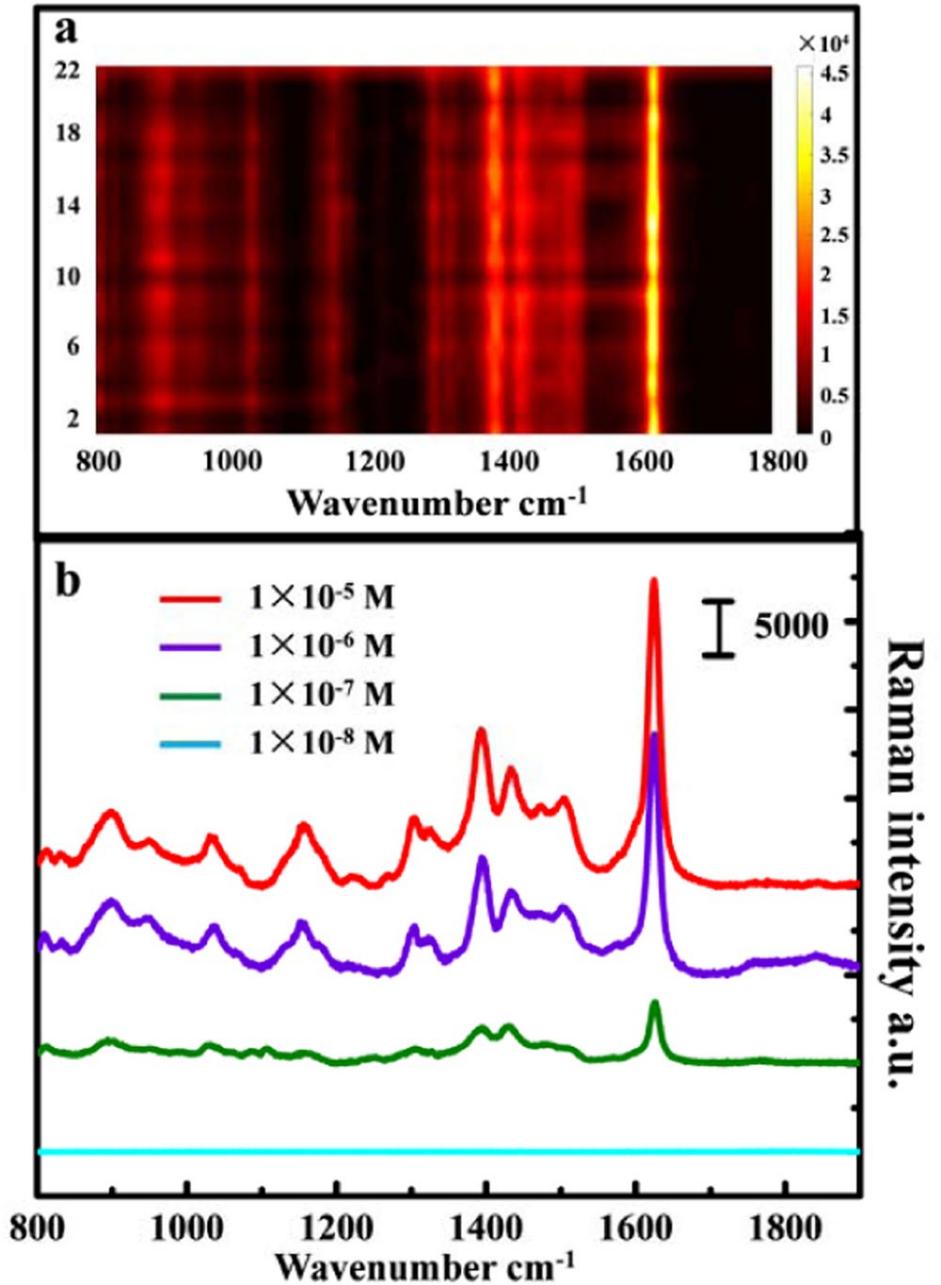
(**a**) The SERS map of MB from different collection positions. (**b**) Raman intensity of MB with different concentrations of 1 × 10^−5^ M, 1 × 10^−6^ M, 1 × 10^−7^ M, and 1 × 10^−8^ M.

In general, an excellent SERS substrate can produce repeatable SERS signals. In this paper, we used MB molecules as probe molecules to detect the SERS performance of microfluidic substrates. To investigate the SERS reproducibility of the prepared substrate, 23 points were randomly selected on the same substrate to collect the signal of methylene blue molecule (1 × 10^−5^ M), and the SERS map of MB from different collection positions is shown in Fig. 5a. The results show that the Raman intensity of the molecules selected at 23 points randomly is similar. It is indicated that the surface properties and structure of the microfluidic substrate are relatively uniform. Therefore, the reproducibility of the SERS signal is excellent. The above results indicate that the method of microfluidic SERS substrate we manufactured greatly overcomes the disadvantage of unevenness compared to substrates that are continuously injected into the Ag precursor synthesis. Different concentrations of methylene blue solution were passed into the microfluidic SERS substrate we made, and the Raman spectrum was collected in Fig. 5b. The results show that the SERS signal of the methylene blue molecule was weakened as its concentration decreased. The detection limit of the MB molecule was 1 × 10^−7^ M.

## Conclusions

In summary, a reliable synthetic protocol of uniform NPs is reported in this work. By comparison experiments, heating for 8 minutes and heating four times can obtain a better SERS active substrate. The high-resolution SEM and uniform SERS signal images demonstrated that the final product was uniform. All in all, in this paper, a simple, fast method for fabricating SERS activity substrate possesses a great scientific value and can be used in lots of fields.

## Methods

### Materials

Silver nitrate (AgNO_3_, 99.99%), ethylene glycol (EG), PVP, copper (II) chloride (CuCl_2_), methylene blue (MB), and ethanol were purchased from Shanghai Aladdin biochemical Polytron Technologies Inc. (Shanghai, China). As-purchased Teflon capillary and syringes were directly used, which is widely commercially available online. High-purity deionized water (18.25 MΩ·cm) was produced using Aquapro AWL-0502-H (Aquapro International Company LLC., Dover, Delaware). After the glassware was thoroughly cleaned with deionized water, it was used for experiments. Polydimethylsiloxane (PDMS) monomer Sylgar 184 and curing agents were purchased from Dow Corning (USA).

### Chip fabrication

PDMS stamps were designed with AutoCAD software and printed on a silicon wafer. Briefly, the PDMS precursor and curing agent were thoroughly mixed in a 10:1 ratio and degassed for 30 minutes before pouring into a pre-casted mold^33–35^. Curing was carried out in a drying oven at 70 °C for 4 h for the polymer to cure. The cured PDMS sheet was carefully detached from the mold after cooling to room temperature and cut into appropriate patterns. The surface of the punched PDMS sheet with the microchannel patterns and glass slide was treated with plasma oxidation for 30 s to make them permanently bound. The size of the microfluidic channel was 200 μm wide and 14 mm long.

### Characterization

The surface morphology of the AgNPs structures was obtained by a scanning electron microscope (SEM, Mira 3 FE, Tescan). The numerical simulation of the NPs is studied by using Finite-Difference Time-Domain (FDTD) method. The optical properties were characterized by SERS spectroscopy, and the SERS signals of target materials were collected with the Raman spectrograph with a 633 nm He-Ne laser (10 mW). The signals were obtained with one scan every 20 s in all measurements.
